# Fight or Flee: An Interesting Case of Snakebite With Delayed Recovery

**DOI:** 10.7759/cureus.20280

**Published:** 2021-12-08

**Authors:** Gunasri Kadirvelu, Kothai Gnanamoorthy, Prasanna Karthik Suthakaran

**Affiliations:** 1 Anaesthesiology, SRM Medical College Hospital and Research Centre, SRM Institute of Science and Technology, Chennai, IND; 2 Internal Medicine, SRM Medical College Hospital and Research Centre, SRM Institute of Science and Technology, Chennai, IND; 3 Internal Medicine, Saveetha Medical College Hospital and Research Institute, Kancheepuram, IND

**Keywords:** mechanical ventilation, delayed recovery, respiratory failure, neurotoxicity, krait, snakebite

## Abstract

Snakebite is a neglected tropical disease, which is very common in the Indian subcontinent. The severity of respiratory muscle paralysis and the delay in recovery depend upon the dose of the venom injected, the severity of the venom, the species of the snake, the duration of presentation to the hospital, and the time and dose of administration of anti-snake venom (ASV). The reasons for this delayed neuromuscular recovery still remain an enigma. We highlight such a case of a young adult who had delayed neuromuscular recovery and prolonged ventilatory support following a neurotoxic snakebite.

## Introduction

The World Health Organization refers to snakebite envenomation as a “neglected tropical disease” [1]. Snakebites are very common in the Indian subcontinent and Southeast Asia. The mortality and morbidity associated with the disease have always been underestimated since many of the patients do not report the snakebites to the authorities [2,3]. The data from the Million Death Study revealed that snakebite envenomation might be responsible for as much as 45,900 deaths annually. It also revealed that most of the deaths happen in rural India where access to primary healthcare is limited and that the monsoon season had the highest incidence of these deaths [4].

The management of neurotoxic envenomation depends on the clinical manifestations of the patient. The simple neurotoxic manifestations of muscular paralysis may recover with the use of polyvalent anti-snake venom (ASV). The involvement of the respiratory and bulbar muscles may necessitate the additional use of ventilatory support [5]. The duration of recovery is also varied, with some patients recovering within 24-48 hours of treatment, while other patients require prolonged ventilatory support. Some patients can also present with a delayed onset of neurological symptoms, which can mimic Guillain-Barré syndrome (GBS) and critical illness polyneuropathy (CIP), and also require a longer time for recovery [6-9].

Here, we report a case of an 18-year-old male who presented with features of neurotoxic snakebite, had a long duration of hospital stay and prolonged ventilatory support, and had a delayed neuromuscular recovery.

## Case presentation

**Figure 1:**
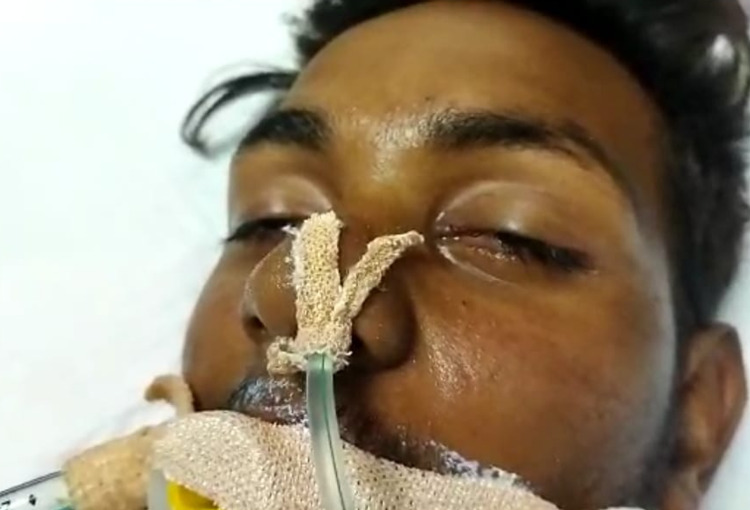
Ptosis with respiratory paralysis

An 18-year-old young male, a construction worker by occupation, was brought to the emergency department by his friends around 4 am; he has a history of snakebite (common krait) on his right hand around 2 am while he was sleeping. The patient subsequently developed swelling of his right hand and forearm, followed by multiple episodes of vomiting. He became drowsy subsequently. He was brought to the hospital after receiving first aid at a nearby clinic. At the time of admission, the fang mark was noted on the dorsal aspect of the right hand near the base of the thumb. He was drowsy (Glasgow Coma Scale: 8/15), tachycardic (124 beats/minute), tachypneic (32 breaths/minute), hypertensive (180/110 mm Hg), and hypoxemic (SpO_2_: 60% on room air, PaO_2_: 54 with 15 L of O_2_) with severe respiratory acidosis (pH: 7.05, pCO_2_: 84 mm Hg). Neurological examination revealed flaccid quadriparesis with bilateral ptosis and bilateral external ophthalmoplegia with pupils sluggishly reacting to light. Other system examinations were unremarkable. In view of his worsening respiratory failure, he was emergently intubated and started on mechanical ventilation (Figure 1).

His basic blood investigations were within normal limits. His 20-minute whole blood clotting time did not reveal any coagulation abnormalities. He was given an initial dose of 10 vials of polyvalent ASV (Bharat Serum, each vial neutralizing 0.6 mg of cobra (Naja naja) venom, 0.45 mg of common krait (*Bungarus caeruleus*) venom, 0.6 mg of Russell's viper (*Vipera russelli*) venom, and 0.45 mg of saw-scaled viper (*Echis carinatus*) venom) as an intravenous infusion in normal saline over 30 minutes, and since there was no improvement in his neurological status, a further 10 vials of polyvalent ASV were given over another 30 minutes in a similar fashion. At this point, the patient was transferred to the intensive care unit for further management. Detailed neurological examination was done, and the patient was found to have dilated pupils with complete ophthalmoplegia. Deep tendon reflexes were absent, and there was no response to painful stimuli. The patient was on assist volume control mode with no trigger activity on the ventilator.

**Figure 2:**
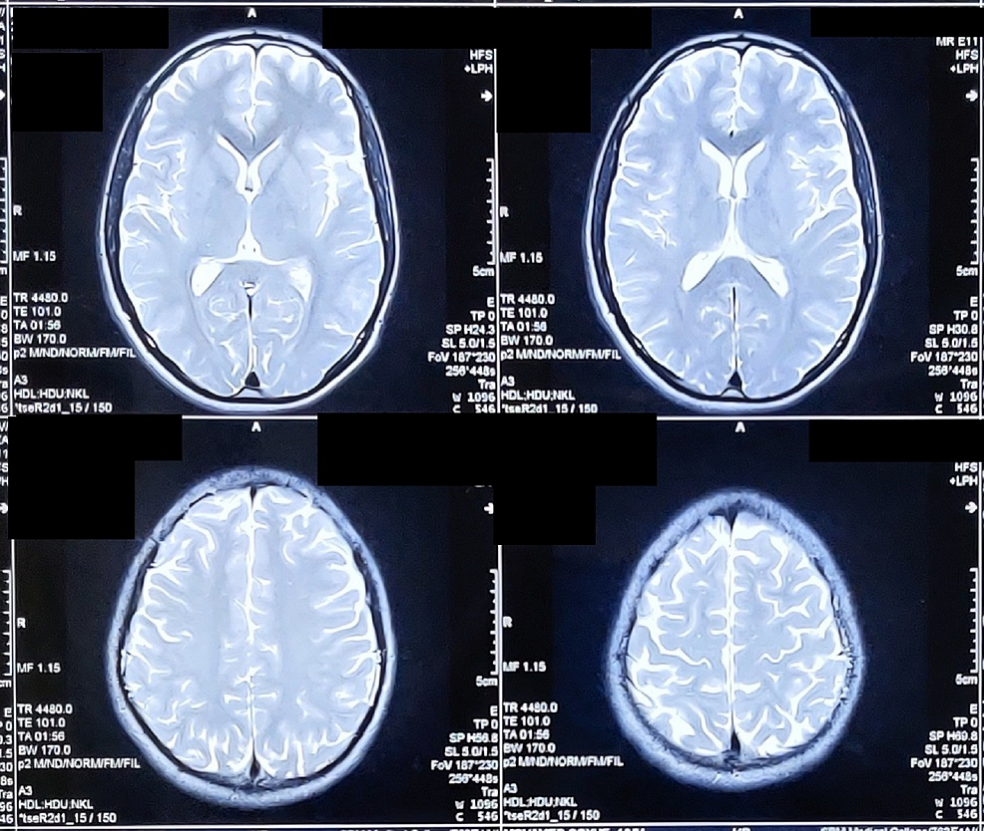
T2-weighted MRI of the brain showing normal study

On day 2 of hospital admission, in view of the delayed neurological recovery, the patient underwent magnetic resonance imaging (MRI) of the brain to rule out any intracranial pathology after discussion with the neurologist. His MRI was normal (Figure 2). As there was no history of preceding fever and no meningeal signs were present, CSF analysis was deferred. The possibility of organophosphorus poisoning was considered in view of the flaccid paralysis. It was ruled that his serum pseudocholinesterase was 6735 U/L (normal range: 4600-11500 U/L). The patient continued to be in a state of deep unarousable coma even after 48 hours of envenomation. Persistent sympathetic overactivity (sinus tachycardia and hypertension) was present throughout the period.

On the fourth day, he started to show minimal response to painful stimuli. Other possible diagnoses were also considered. CSF analysis done on day 4 revealed no abnormal findings. Multiple attempts for weaning from ventilatory support were unsuccessful in view of his persistent muscle weakness. In view of his prolonged requirement of ventilatory support, he underwent tracheostomy on day 8 and was continued on mechanical ventilation. On day 12 of the hospital stay, he developed ventilator-acquired pneumonia. The tracheal aspirate culture showed mixed growth of *Klebsiella pneumoniae* and *Pseudomonas aeruginosa* for which he was treated with intravenous meropenem for 10 days.

**Figure 3:**
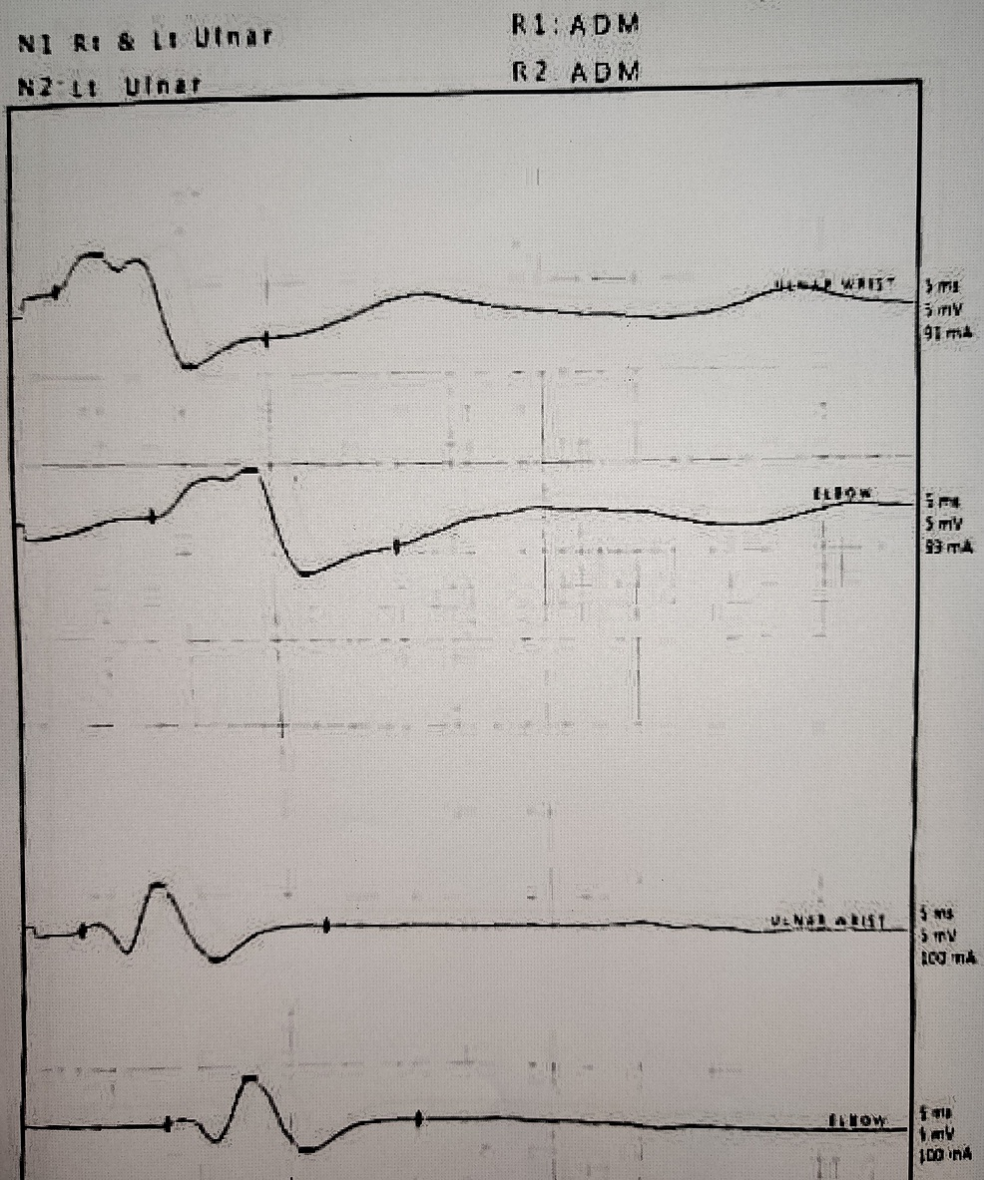
Nerve conduction study of the ulnar nerve showing increased sensory latency and reduced SNAPs

As he continued to have a persistent neuromuscular weakness (power: grade 3 in all four limbs, diminished deep tendon reflexes), a nerve conduction study was done on day 16. It showed bilateral sensory and motor axonal types of polyneuropathy with an increase in the sensory latency of the bilateral median and ulnar nerve. Bilateral median and superficial peroneal nerve sensory nerve action potentials (SNAPs) were also reduced (Figure 3).

On day 24 of his hospital stay, he was finally weaned off the ventilator and was on room air maintaining adequate saturation. He continued to have muscle weakness and was being continued on physiotherapy. He was shifted out from the ICU on day 27 and was treated in the ward. On day 35, the patient underwent closure of the tracheostomy. He was discharged from the hospital on day 40, at which time he had an increase in his power (grade 4+ in all four limbs). He was advised for a regular follow-up.

## Discussion

The common venomous snakes in India belong to the cobra species and krait species, which are members of the Elapidae family [5]. The envenomation by these snakes results in neurotoxicity. Neurotoxicity can lead to acute neuromuscular paralysis of all muscles, including the bulbar and respiratory muscles, and direct toxicity to the nerves. The variations in the clinical presentations of patients with neurotoxic envenomation have been attributed to the variations in the constituent toxins of the venom across seasons, geographical areas, and genetic difference within the species. In patients who have a prolonged course of manifestations due to snakebite, various possibilities could be possible reasons, such as the dose of the venom injected, the makeup of the venom injected, the delay in presentation to the hospital, and the dose of the ASV administered [10].

The venom of Elapidae contains neurotoxins that are classified into alpha and beta bungarotoxins. Alpha bungarotoxins act postsynaptically, whereas beta toxins act presynaptically [11]. Beta bungarotoxins contain predominantly phospholipase A2, which can lead to the depletion of synaptic vesicles from the motor nerve terminals of the skeletal muscle, followed by the destruction of the motor nerve terminal and the degeneration of the cytoskeleton of the intramuscular axons by disrupting the phospholipids present in the cell membranes [12]. This can possibly explain the axonopathy pattern found in the nerve conduction study in our patient. The destruction of the motor terminal is usually irreversible, and clinical recovery of the patient depends on the regeneration of new nerve terminals, which can contribute to the varying length of respiratory paralysis in patients [13]. It is also reported in the literature that in patients with delayed recovery, there were significant changes, such as prolongation of sensory, motor, and f-wave latencies and reduction in conduction velocities, which was attributed to a direct systemic effect of the envenomation [14].

In a large study from Sri Lanka, Kularatne reported a clinical series of 210 krait bites. In these patients, autonomic dysfunction leading to tachycardia was present in about 188 patients, and 35 of those patients had a dilated and fixed pupil resembling coma, similar to the patient in this study. Also, the maximum duration of respiratory paralysis reported in the study was 29 days, which was similar to this patient [6].

With the use of polyvalent ASV, the circulating venom is usually neutralized. However, the already bound toxin is not neutralized and can continue to act. This can explain the reason for the failure of all patients to respond adequately to the maximum dose of ASV. It is in this regard that early and timely administration of ASV can make a difference in the outcomes of patients who have snakebite envenomation [14]. The use of acetylcholinesterase inhibitors to reverse the neuromuscular blockade due to snake envenomation is useful only with postsynaptic toxins and not with presynaptic toxins [10].

## Conclusions

This case is presented to highlight the unusual and delayed recovery of a patient from a snakebite, possibly the common krait. In spite of the adequate and early use of ASV, the patient still had a delayed recovery and continued to have a slow recovery. The case throws some possible light on some of the long-term effects of the neurotoxins present in snake venom, namely, phospholipase A2, which can cause direct damage to the nerve terminals and not a simple interruption of the neuromuscular transmission. It is necessary to be aware of the delayed neurological issues while treating a patient with a neurotoxic snakebite. Although some patients may mimic other neurological conditions such as locked-in state, it is necessary to remember the possibility of snake envenomation in such patients, especially when there is no clear history available.
